# *In vitro* interaction of artemisinin derivatives or the fully synthetic peroxidic anti-malarial OZ277 with thapsigargin in *Plasmodium falciparum* strains

**DOI:** 10.1186/1475-2875-12-43

**Published:** 2013-01-31

**Authors:** Oyindamola O Abiodun, Reto Brun, Sergio Wittlin

**Affiliations:** 1 Department of Pharmacology and Therapeutics, University of Ibadan, Ibadan, Nigeria; 2 Malaria Research Laboratories, College of Medicine, University of Ibadan, Ibadan, Nigeria; 3 Parasite Chemotherapy Unit, Swiss Tropical and Public Health Institute, CH-4002, Basel, Switzerland; 4 University of Basel, CH-4003, Basel, Switzerland

**Keywords:** Thapsigargin, Artesunate, Artemether, OZ277, *Plasmodium falciparum*, Interaction study, Isobolograms

## Abstract

**Background:**

Semi-synthetic artemisinin derivatives are powerful peroxidic drugs in artemisinin-based combination therapy (ACT) recommended as first-line treatment of *Plasmodium falciparum* malaria in disease-endemic countries. Studies by Eckstein-Ludwig and co-workers showed both thapsigargin and artemisinin specifically inhibit the sarcoplasmic reticulum Ca^2+^−ATPase of *Plasmodium falciparum* (PfATP6). In the present study the type of interaction between thapsigargin and artemisinin derivatives as well as the ozonide OZ277 (RBx11160 or arterolane) was evaluated in parasite cultures. The latter compound is an adamantane-based peroxide and the first fully synthetic clinical candidate recently registered in India by Ranbaxy Laboratories Ltd. for anti-malarial combination therapy.

**Methods:**

Drug interaction studies were performed using a previously described fixed ratio method and anti-malarial activity measured using the [^3^H] hypoxanthine incorporation assay.

**Results:**

The sum 50% and 90% fractional inhibitory concentration (∑FIC_50, 90_) of the interaction of thapsigargin with OZ277, artemether or artesunate, against NF54 and K1 strains of *P. falciparum* ranged from 0.9 to 1.4.

**Conclusion:**

The interaction of thapsigargin with OZ277, artesunate or artemether was additive, data consistent with previous observations indicating that activity of anti-malarial peroxides does not derive from reversible interactions with parasite targets.

## Background

Artemisinin is a sesquiterpene lactone isolated from *Artemisia annua*. Semi-synthetic artemisinin derivatives are important endoperoxides in artemisinin-based combination therapy (ACT) recommended as first-line treatment of *Plasmodium falciparum* malaria in endemic countries. Despite their importance, the precise mechanism of action of this compound class is still under debate. The dominating view for all the peroxides (artemisinin derivatives and synthetic peroxides such as ozonides) is that they undergo reductive activation of the peroxide in the presence of ferrous iron released upon haemoglobin digestion within the parasite food vacuole [1]. This forms carbon centred radicals, which then alkylate key parasite proteins like haem and membrane-associated parasite proteins [2], one of which is the translationally controlled tumour protein (PfTCTP) [3] and another could be PfATP6, a SERCA-type Ca^2+^-ATPase [4]. Studies by Eckstein-Ludwig and co-workers showed that thapsigargin and artemisinin specifically inhibit PfATP6 with Ki values (half-maximum inhibition constants) of 146 and 162 nM, respectively [4]. In addition, artemisinin was shown to have a similar distribution in parasites when compared with thapsigargin, a specific inhibitor of PfATP6. Also, interaction between thapsigargin and artemisinin resulted in antagonism in cultured parasites. Interestingly, Crespo and co-workers reported that artemisinin and two novel epoxy-endoperoxides showed additive activity when used in combination with thapsigargin [5]. In the present study the type of interaction between thapsigargin and artemisinin derivatives as well as the ozonide OZ277 (RBx11160 or arterolane) [6,7] was evaluated in parasite cultures for the first time. A previous study to understand the mechanism(s) of action of OZ277 revealed that it inhibits PfATP6 with a Ki value of 7,700 nM; thus it is a much weaker inhibitor than artemisinin [8]. The study further showed that a fluorescent derivative of OZ277 displayed patterns of subcellular distribution different from that of artemisinin [8]. In order to shed more light on the mechanism of action of peroxidic anti-malarials vis à vis their interaction with PfATP6 in cultured parasites, the interaction of thapsigargin with OZ277 or the semi-synthetic artemisinin derivatives (artesunate and artemether) was evaluated in *Plasmodium falciparum* K1 and NF54 strains.

## Methods

### Compounds

The compounds used in this study were from the following sources: OZ277 tosylate (J L Vennerstrom, Nebraska, USA), artemether (Kunming Pharmaceuticals Corp, China), artesunate (Roche, Basel, Switzerland) and thapsigargin (Sigma, USA).

### *In vitro* anti-malarial activity

*Plasmodium falciparum* strains NF54 (sensitive to all known anti-malarial drugs) and K1 (chloroquine/pyrimethamine resistant) were cultivated and maintained in continuous culture using standard techniques [9,10]. Initial concentrations for test compounds were 10 ng/ml for artemether, artesunate, OZ277 and 20,000 ng/ml for thapsigargin. Two-fold serial dilutions were done in hypoxanthine-free culture medium in 96-well plates over a 64-fold range, the lowest being 0.16 ng/ml and 313 ng/ml for the anti-malarial drugs and thapsigargin respectively. One hundred μl of an asynchronous parasite culture (0.3% parasitaemia and 2.5% haematocrit) was added to the drug solutions (100 μl volume) in 96-well plates. After 48-hr incubation, 0.5 microCi of ^3^H]hypoxanthine (Anawa, Zürich, Switzerland) in 50 μl of medium was added to each well and plates were incubated for an additional 24 hr. Parasites were harvested onto glass-fibre filters and radioactivity was counted using a Betaplate liquid scintillation counter (Wallac, Zurich). The results were recorded as counts per minute per well at each drug concentration and expressed as a percentage of the untreated controls. Fifty percent inhibitory concentrations (IC_50_) were estimated by linear interpolation [11].

### *In vitro* drug interactions

Drug interaction studies were performed using a modification of the fixed ratios method [9,12]. Initially, the 50% inhibitory concentration (IC_50_) values of the individual test compounds were determined. Subsequently, the anti-malarial compounds and thapsigargin were diluted with hypoxanthine-free culture medium to initial concentrations of 10 times the predetermined IC_50_ and the solutions combined in ratios of 1:3, 1:1, and 3:1. One hundred μl of the individual test compounds and mixtures were then introduced into the 96-well plates to give duplicate columns. The IC_50_ of the test compounds alone and in combination were determined as described above. For data interpretation, the IC_50_ or IC_90_ of the drugs in combination were expressed as fractions of the IC_50_ or IC_90_ of the individual drugs. These fractions were called fractional inhibitory concentrations (FIC) for drug A and for drug B, respectively.

### Interpretation of isobolograms

Isobolograms were constructed by plotting the FIC of drug A (FIC_A_) against the FIC of drug B (FIC_B_) for each of the three ratios, with concave curves indicating synergy, straight lines indicating addition and convex curves indicating antagonism. To obtain numeric values for the interactions, results were expressed as the sum FICs (∑FICs) of the FIC_A_ and FIC_B._ Cutoff ranges were determined by mixing the same drug at various ratios and accounting for experimental variation. ∑FIC values indicate the nature of the interactions as follows: ∑FIC<0.8 is synergistic, ∑FIC 0.8 to 1.4 is additive, ∑FIC>1.4 is antagonistic. Mean ∑FICs were used to classify the overall nature of the interaction.

## Results and discussion

The susceptibility profile of OZ277, artesunate, artemether and thapsigargin was determined against both chloroquine-sensitive and -resistant strains of *P. falciparum* (NF54 and K1) before embarking on the interaction studies. The two groups of peroxides displayed potent anti-malarial activity with IC_50_ and IC_90_ values ranging from 0.17 to 0.98 ng/ml and 0.42 to 1.9 ng/ml respectively (Table 1). In contrast thapsigargin showed anti-malarial activity that is approximately three to four log orders less potent (IC_50_ range of 1,600-1,900 ng/ml). Similar IC_50_ values for thapsigargin in parasite culture have also been reported previously [4].

**Table 1:** ***In vitro *anti**-**malarial activity of semi**-**synthetic artemisinins**, **synthetic OZ277 and thapsigargin against *Plasmodium falciparum *K1 and NF54 strains**

**Compounds**	**Inhibitory concentrations (ng/ml)**			
	^**+**^***P. falciparum *K1**		^**+**^***P. falciparum *NF54**	
	**(Mean ± SE)**		**(Mean ± SE)**	
	**IC**_**50**_	**IC**_**90**_	**IC**_**50**_	**IC**_**90**_
Artesunate	0.60 ± 0.18	1.8 ± 0.1	0.98 ± 0.48	1.9 ± 1.1
Artemether	0.45 ± 0.28	0.80 ± 0.34	0.49 ± 0.18	0.98 ± 0.27
OZ277	0.17 ± 0.03	0.42 ± 0.11	0.82 ± 0.09	1.6 ± 0.4
Thapsigargin	1612 ± 140	6850 ± 730	1940 ± 433	7455 ± 2307

^+^Data was obtained from three independent experiments. SE = standard error.

Subsequently, the *in vitro* interaction of OZ277 and semi-synthetic artemisinin derivatives with thapsigargin against *P. falciparum* strains with different degrees of chloroquine sensitivity was evaluated. The employed fixed ratio isobologram method was described earlier by Fivelman and co-workers [12] and has been validated using a series of *in vitro* control combination assays [9]. These control assays include: *in vitro* interaction of artemether with pyrimethamine (proven antagonistic) [13,14] and atovaquone with proguanil (proven synergistic) [15,16]. Interactions data were analysed at the IC_50_ and IC_90_ levels.

The sum 50% and 90% fractional inhibitory concentration (∑FIC_50, 90_) of the interaction of thapsigargin with OZ277, artemether or artesunate, against both strains of *P. falciparum* ranged from 0.9 to 1.4 at the three ratios (1:3, 1:1 and 3:1 tested, Table 2, Additional file 1). This additive interaction is not unexpected based on the lack of antagonism observed between either artemisinin or OZ277 and their non-peroxide isosteres, data demonstrating that the activity of these drugs does not derive from reversible interactions with parasite targets [17]. Such additive interactions are also in line with data published by Crespo and co-workers [5], who demonstrated that artemisinin and two novel endoperoxides show additive activity when used in combination with thapsigargin. In contrast, data from Eckstein-Ludwig *et al.*[4] showed that the combination of artemisinin and thapsigargin was antagonistic (∑FIC 1.5-5.0), which is possible in a scenario in which artemisinin and thapsigargin inhibit PfATP6 reversibly (non-covalent modification) [18].

**Table 2:** ***In vitro *interaction of thapsigargin with OZ277 or semi-synthetic artemisinin derivatives against *Plasmodium falciparum *K1 and NF54 strains**

**Thapsigargin combined with:**	***P. falciparum *strains**	^**+**^**(∑FIC) ± Standard error at different ratio of drug combinations**							
		**1:3**		**1:1**		**3:1**			
		∑FIC_50_	∑FIC_90_	∑FIC_50_	∑FIC_90_	∑FIC_50_	∑FIC_90_	mean ∑FIC_50_*	mean ∑FIC_90_*
OZ277	K1	1.0 ± 0.0	1.0 ± 0.1	1.1 ± 0.0	1.1 ± 0.1	1.4 ± 0.1	1.4 ± 0.1	1.2	1.2
	NF54	1.0 ± 0.1	1.0 ± 0.0	1.2 ± 0.1	0.9 ± 0.0	1.1 ± 0.1	0.9 ± 0.0	1.1	0.9
Artemether	K1	1.2 ± 0.2	1.1 ± 0.0	1.1 ± 0.0	1.0 ± 0.1	1.1 ± 0.1	0.9 ± 0.1	1.1	1.0
	NF54	1.1 ± 0.1	1.0 ± 0.1	0.9 ± 0.1	0.9 ± 0.0	1.2 ± 0.1	1.0 ± 0.0	1.1	1.0
Artesunate	K1	1.2 ± 0.0	1.2 ± 0.0	1.2 ± 0.1	1.2 ± 0.2	1.3 ± 0.0	1.1 ± 0.1	1.2	1.2
	NF54	1.1 ± 0.0	1.0 ± 0.0	1.1 ± 0.1	1.0 ± 0.1	1.0 ± 0.0	1.0 ± 0.1	1.1	1.0

^+^ Data was obtained from three independent experiments. * m∑FICs was used to classify the overall nature of the interaction which appears to be additive in this study.

## Conclusion

The interaction of thapsigargin with OZ277, artesunate or artemether was additive, data consistent with previous observations indicating that activity of anti-malarial peroxides does not derive from reversible interactions with parasite targets.

## Abbreviations

OZ277: Ozonide OZ277 (RBx11160 or arterolane); PfTCTP: Plasmodium falciparum translationally controlled tumour protein; PfATP6: Plasmodium falciparum Ca2+ − ATPase; ∑FICs: sum of fractional inhibitory concentration; NF54: Plasmodium falciparum strain (sensitive to all known anti-malarial drugs); KI: chloroquine/pyrimethamine resistant strain of Plasmodium falciparum; SERCA-type Ca^2+^-ATPase: Sarcoplasmic reticulum Ca2+ − ATPase; FIC_A_: fractional inhibitory concentration (FIC) for drug A; FIC_B_: fractional inhibitory concentration (FIC) for drug B.

## Competing interests

The authors declare that they have no competing interests.

## Authors’ contributions

OOA contributed to design, acquisition of data, analysis and interpretation of data. SW contributed to conception, design of the study and interpretation of data. OOA, RB and SW were involved in drafting the manuscript or revising it critically for important intellectual content. All authors read and approved the final manuscript.

## Supplementary Material

Additional file 1 **Representative isobolograms of in vitro interactions of thapsigargin with OZ277 or semi-synthetic artemisinins against *Plasmodium falciparum*.** Click here for file
