# Supplementary material for: In vitro interaction of artemisinin derivatives or the fully synthetic peroxidic anti-malarial OZ277 with thapsigargin in Plasmodium falciparum strains
Source: Malar J. 2013 Jan 31;12:43. doi: 10.1186/1475-2875-12-43 (PMC3566918; doi:10.1186/1475-2875-12-43)
Supplement: Additional file 1 — Representative isobolograms of in vitro interactions of thapsigargin with OZ277 or semi-synthetic artemisinins against Plasmodium falciparum. [file 1475-2875-12-43-S1.pdf]

Supplementary Figure 1:
Representative isobolograms of in vitro interactions of thapsigargin with OZ277 or semi-synthetic artemisinins against *Plasmodium falciparum* NF54

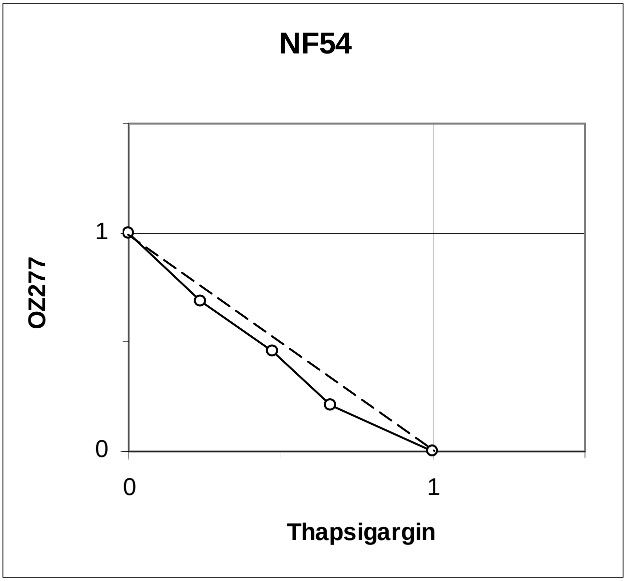

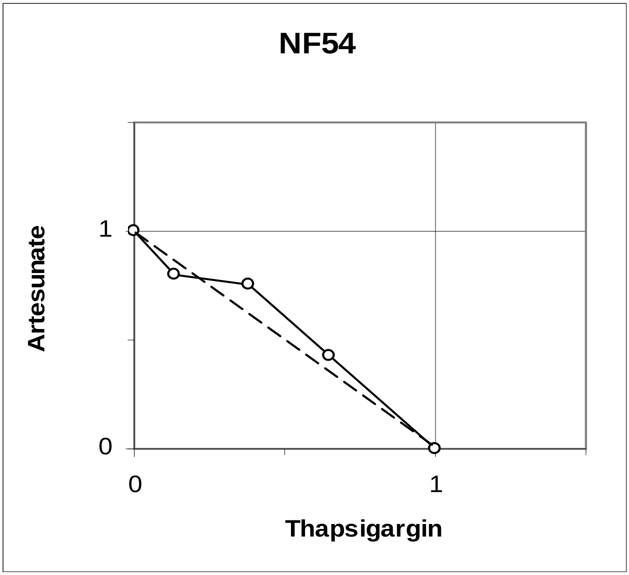

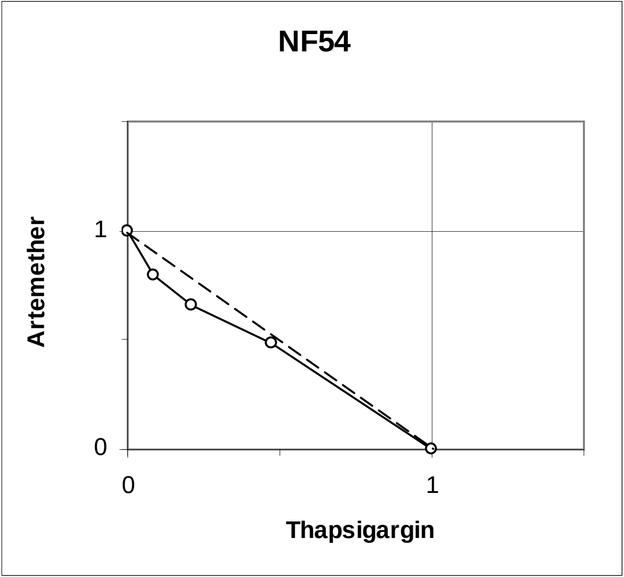
NF54
Artemether
1
0
0
1
Thapsigargin
